# The rate of glycolysis quantitatively mediates specific histone acetylation sites

**DOI:** 10.1186/s40170-015-0135-3

**Published:** 2015-09-23

**Authors:** Ahmad A. Cluntun, He Huang, Lunzhi Dai, Xiaojing Liu, Yingming Zhao, Jason W. Locasale

**Affiliations:** Graduate Field of Biochemistry, Molecular Cell Biology, Cornell University, Ithaca, NY USA; King Abdullah International Medical Research Center (KAIMRC), Riyadh, Saudi Arabia; Ben May Department of Cancer Research, The University of Chicago, Chicago, IL USA; Division of Nutritional Sciences, Cornell University, Ithaca, NY USA; Department of Pharmacology and Cancer Biology, Duke University Medical School, Durham, NC USA; Duke Cancer Institute, Duke University Medical School, Durham, NC USA; Duke Molecular Physiology Institute, Duke University Medical School, Durham, NC USA

**Keywords:** Acetyl coenzyme A (acetyl***-***CoA), Histone acetylation, Warburg effect, Glycolysis, Cancer

## Abstract

**Background:**

Glucose metabolism links metabolic status to protein acetylation. However, it remains poorly understood to what extent do features of glucose metabolism contribute to protein acetylation and whether the process can be dynamically and quantitatively regulated by differing rates of glycolysis.

**Results:**

Here, we show that titratable rates of glycolysis with corresponding changes in the levels of glycolytic intermediates result in a graded remodeling of a bulk of the metabolome and resulted in gradual changes in total histone acetylation levels. Dynamic histone acetylation levels were found and most strongly correlated with acetyl coenzyme A (ac-CoA) levels and inversely associated with the ratio of ac-CoA to free CoA. A multiplexed stable isotopic labeling by amino acids in cell culture (SILAC)-based proteomics approach revealed that the levels of half of identified histone acetylation sites as well as other lysine acylation modifications are tuned by the rate of glycolysis demonstrating that glycolytic rate affects specific acylation sites.

**Conclusions:**

We demonstrate that histone acylation is directly sensed by glucose flux in a titratable, dose-dependent manner that is modulated by glycolytic flux and that a possible function of the Warburg Effect, a metabolic state observed in cancers with enhanced glucose metabolism, is to confer specific signaling effects on cells.

## Background

The metabolism of carbohydrates through glycolysis affects numerous cellular processes through its effects on biosynthetic, energy, and reactive oxygen species metabolism. Glucose metabolism is altered in pathological conditions such as cancer and autoimmunity [1, 2]. In these cases, cells metabolize their glucose, in oxygenated conditions, at higher rates and ferment the glucose to produce lactate that together is referred to as the Warburg effect or aerobic glycolysis [3]. There are many hypotheses for the biological function of the Warburg effect [4, 5]. It has been proposed that since glycolysis to lactate occurs at a faster rate than that of oxidative phosphorylation, glycolysis provides a means to generate ATP more rapidly [6, 7]. It has also been proposed that the Warburg effect is an adaptation to support the biosynthetic requirements of cancer cells [8, 9]. Each of these proposals however is not without difficulties. For example, estimates have found that over 90 % of the carbon from glucose is secreted as lactate and alanine, leaving little room for biosynthesis [10]. Also, the rapid ATP produced by glycolysis can also be obtained by other mechanisms such as creatine kinase and adenylate kinase [11, 12].

Additionally, direct biochemical signaling functions of glucose metabolism are also possible [13, 14]. When the rate of glycolytic flux changes, it is possible that the levels of metabolites that serve as intermediates in glycolysis and associated pathways are consequentially altered. Changes in metabolite levels, if they are used as substrates or cofactors for enzymes that carry out posttranslational modifications (PTMs), could allow for metabolism to confer an active role in cell physiology thus conferring signaling functions [4, 15–17]. For example, if these PTMs affect the conformation of chromatin, they can potentially alter the expression of thousands of genes.

Numerous studies have shown that changes in glucose metabolism can regulate histone acetylation [18–25]. There are multiple molecular links from glucose metabolism to protein acetylation status including intracellular pH [26]. pH is mediated by lactate, the product of aerobic glycolysis. Acetyl coenzyme A (ac-CoA), a product of glycolysis is the substrate used by acetyltransferases to modify transfer of the ac-CoA moiety to histones. In addition, the free coenzyme A (CoA) product can inhibit these enzymes [27]. Also, the redox status mediated by NAD^+^ and NADH can affect the activity of certain deacetylases [28]. Furthermore, ketone bodies, which are produced during fatty acid oxidation, can also inhibit histone deacetylases [28, 29]. Despite these intriguing biochemical links, it remains unknown how these phenomena conspire together and how they are quantitatively mediated. Their specificity to directly defined acetylation sites is also poorly understood. Also, multiple factors can contribute to the regulation of histone acetylation from altered glucose metabolism and pinpointing the affecting factors in metabolism that regulates histone acetylation. Moreover, recently discovered histone modifications such as histone propionylation, butyrylation, and 2-hydroxyisobutyrylation have each been identified as unique histone modifications important for gene expression [30–33]. Much like histone acetylation, these modifications use their corresponding ac-CoA metabolite species as substrates for the PTM. How metabolic flux emanating from glucose affects the specific pathways that supply these cofactors for these modifications remains unknown. Furthermore, it remains unclear how coordinated changes in metabolite concentrations at the systems level conspire in the face of altered glucose metabolism to affect biologically important histone acetylation.

To test the hypothesis that glycolysis rate alters the levels of metabolites that in turn regulate histone acetylation, we considered a system where we can quantitatively tune the rate of glycolysis and as a result affect the concentrations of metabolites in glycolysis. We then carried out a metabolomics study that considered how differences in metabolite concentrations quantitatively affect histone acetylation. In measuring each potential contribution to histone acetylation, we found several factors that contribute to histone acetylation. Together, these results provide a quantitative model for the contributions of glycolysis to altered histone acetylation and acylation and provide evidence for the direct sensing of glucose metabolism by histone acetylation. Since histone acetylation controls and in part determines the expression of thousands of genes, it is tempting to speculate that this mechanism may provide a general link from metabolism to the chromatin state in cells.

## Methods

### Cell culture and reagents

HCT116 colorectal cancer cell lines were cultured in full media composed of RPMI 1640, 10 % fetal bovine serum (FBS), 100 U/mL penicillin, and 100 mg/mL streptomycin. All cell lines were obtained as gifts from Lewis Cantley’s laboratory. Cells were cultured in a 37 °C, 5 % CO_2_ atmosphere. For 2-deoxy-D-glucose (2DG) titration experiments, either 2DG (Sigma) was added to the media at the respective concentrations or 0.01 % DMSO (cellgro) for the vehicle (Veh). At the start of each experiment, cells were seeded at a density of 2.2 × 10^6^ cells for 10-cm plates for protein collection or 3 × 10^5^ cells/well in a 6-well plate for metabolite collection and allowed to adhere and grow to 80 % confluence. Cells were then washed with PBS and allowed to incubate in the respective treatments for 6 h. Histones and metabolites were extracted as described below.

### Immunoblotting

Samples were homogenized in Triton extraction buffer (TEB, 0.5 % Triton X 100, 2 mM phenylmethylsulfonyl fluoride (PMSF), 0.02 % NaN3 in PBS) and centrifuged at 2000 rpm for 10 min at 4 °C. Pellets were resuspended in 0.2 N HCl, and histones were acid extracted overnight at 4 °C. Histones were then precipitated in 100 % trichloroacetic acid (Sigma-Aldrich), washed with cold acetone, and allowed to air dry. Pellets were either stored at −20 or dissolved in ddH2O. Histone extracts were loaded onto 12 % SDS-PAGE gels and transferred to polyvinylidene difluoride (PVDF) membranes. Membranes were blocked in 5 % dry milk in TBST and incubated with anti-acetyl-H3K27 (Abcam) 1:2000, anti-acetyl H3 (Millipore) 1:2000, or anti-H3 (Millipore) 1:10000. Horseradish peroxidase-conjugated anti-rabbit (Rockland), 1:10,000, was used as secondary antibodies. Chemiluminescent signals were detected with Clarity Western ECL Detection Kit (Bio-Rad) and imaged using a ChemiDoc MP System (Bio-Rad).

### Metabolite extraction

The procedures for cultured cells are described in previous studies [34, 35]. Briefly, for culture from adherent cells, the media were quickly aspirated and the cells were washed with cold PBS on dry ice. Then, 1 mL of extraction solvent (80 % methanol/water) cooled to −80 °C was added immediately to each well, and the dishes were transferred to −80 °C for 15 min. The plates were removed, and the cells were scraped into the extraction solvent on dry ice. All metabolite extracts were centrifuged at 20,000*g* at 4 °C for 10 min. Finally, each solvent in each sample was evaporated in a speed vacuum for metabolomics analysis. For polar metabolite analysis, the cell extract was dissolved in 15 μL water and 15 μL methanol/acetonitrile (1:1 *v*/*v*) (liquid chromatography-mass spectrometry (LC-MS) optima grade, Thermo Scientific). For CoA metabolite analysis, the cell extract was dissolved in 50 mM ammonium acetate (pH 6.8). The samples were briefly centrifuged, and the supernatants were transferred to liquid chromatography (LC) vials. The injection volume for polar metabolite analysis was 5μL and for coA analysis it was 8μL.

### U-^13^C-glucose labeling

Cells were first cultured in standard RPMI 1640, 10 % FBS, 100 U/mL penicillin, and 100 mg/mL streptomycin media to 70 % confluence. They were then washed with PBS and treated with corresponding 2DG in fresh media as described above and incubated for 6 h. Subsequently, the cells were washed with PBS and corresponding media conditions where glucose is replaced with U-13C6-D-glucose (Cambridge Isotope Laboratories, Inc.) was considered. Metabolites were then extracted at the indicated time points as described in the text from the spent media.

### Liquid chromatography for metabolite analysis

For polar metabolites, an Xbridge amide column (100 × 2.1 mm i.d., 3.5 μm; Waters) is employed, whereas for nonpolar ac-CoA metabolites, a LUNA C18 column (100 × 2.0 mm; phenomenex) is employed on a Dionex (Ultimate 3000 UHPLC) for compound separation at room temperature. For the polar method, mobile phase A is 20 mM ammonium acetate and 15 mM ammonium hydroxide in water with 3 % acetonitrile, pH 9.0, and mobile phase B is acetonitrile. The linear is as gradient as follows: 0 min, 85 % B; 1.5 min, 85 % B, 5.5 min, 35 % B; and 10 min, 35 % B, 10.5 min, 35 % B, 14.5 min, 35 % B, 15 min, 85 % B, and 20 min, 85 % B. The flow rate was 0.15 ml/min, from 0 to 10 min and 15 to 20 min, and 0.3 ml/min from 10.5 to 14.5 min. For the coA method, mobile phase A is water with 5 mM ammonium acetate, and mobile phase B: methanol. Linear gradient is: 0 min, 2 % B; 1.5 min, 2 % B; 3 min, 15 % B; 5.5 min, 95 % B; 14.5 min, 95 % B; 15 min, 2 % B, 20 min, 2 % B. All solvents are LC-MS grade and purchased from Fisher Scientific.

### Mass spectrometry

The Q Exactive MS (Thermo Scientific) is equipped with a heated electrospray ionization probe (HESI), and the relevant parameters are as listed: evaporation temperature, 120 °C; sheath gas, 30; auxiliary gas, 10; sweep gas, 3; and spray voltage, 3.6 kV for positive mode and 2.5 kV for negative mode. Capillary temperature was set at 320 °C, and S-lens was 55. A full scan range from 60 to 900 (m/z) was used. The resolution was set at 70,000. The maximum injection time was 200 ms. Automated gain control (AGC) was targeted at 3,000,000 ions.

### Metabolomics and data analysis

Raw data collected from LC-Q Exactive MS is processed on Sieve 2.0 (Thermo Scientific). Peak alignment and detection are performed according to the protocol described by Thermo Scientific. For a targeted metabolite analysis, the method “peak alignment and frame extraction” is applied. An input file of theoretical m/z and detected retention time of 263 known metabolites is used for targeted metabolites analysis with data collected in positive mode, while a separate input file of 197 metabolites is used for negative mode. M/Z width is set at 10 ppm. The output file including detected m/z and relative intensity in different samples is obtained after data processing. Quantitation and statistics were calculated and visualized with Microsoft Excel, Gene-E, and MetaboAnalyst online software.

### SILAC experiments

Heavy lysine (^13^C_6_^15^N_2_-L-lysine:2HCl), medium lysine (4,4,5,5-D4-L-lysine:2HCl) and RPMI 1640 Media for stable isotopic labeling by amino acids in cell culture (SILAC) (minus L-lysine and L-arginine) were purchased from Cambridge Isotope Laboratories, Inc. Light lysine (L-lysine HCl) and arginine (L-arginine HCl) were purchased from Amresco.

Cells were inoculated into a 6-well plates and allowed to grow for at least six doublings with respect to SILAC media. Media was replenished every 3 days. The cells were then expanded to 15-cm plates till 80 % confluence. The cells were then treated with corresponding 2DG treatments (100uM, 5 mM, Veh) for 6 h. (5 mM, heavy lysine; 100 μM, medium lysine, Veh, light lysine).

### Histone protein extraction and in-solution digestion

The “heavy”-, “medium,”- and “light”-labeled cells were washed twice with pre-chilled PBS and the core histone proteins were prepared using an acid extraction procedure previously described [36], respectively. Each acid-extracted histone protein solution was clarified by centrifugation for 10 min at 20,000*×g*. The protein concentrations of the supernatants were measured, and equal amounts of proteins from the three pools of cells were combined. Then trypsin (Promega Corp., Madison, WI) was added into the protein mixtures at a trypsin-to-protein ratio of 1:50 (*w*/*w*) for digestion at 37 °C for 16 h.

### Immunoprecipation of post-translational modifications

To enrich the peptides with specific PTMs (lysine acetylation, lysine propionylation, lysine butyrylation, and lysine 2-hydroxyisobutyrylation), 200 μg of tryptic peptides were incubated with PTM-specific antibody-immobilized beads (containing 20 μg of antibody, PTM Biolabs, Chicago, IL) in NETN buffer (50 mM Tris pH 8.0, 100 mM NaCl, 1 mM EDTA, 0.5 % NP40) at 4 °C for 6 h with gentle rotation. The beads were carefully washed three times with NETN buffer, twice with ETN buffer (50 mM Tris pH 8.0, 100 mM NaCl, 1 mM EDTA), and once with ddH_2_O. After immunoaffinity purification (IP), 24.22 % (on average) of the total identified peptides are modified. The bound peptides were eluted from the beads with 0.1 % TFA in water and vacuum-dried. The resulting peptides were dissolved in 0.5 % formic acid-water solution and desalted with Ziptip C18 (EMD Millipore Corp., Darmstadt, Germany) according to the manufacturer’s instructions.

### Analysis of tryptic peptides

The desalted samples were dissolved in 2.5 μL HPLC A buffer containing 0.1 % formic acid and 99.9 % water (*v*/*v*). The solution was directly loaded onto a homemade capillary column (10 cm length with 75 μm inner diameter) packed with Jupiter C12 resin (4 μm particle size, 90 Å pore size, Phenomenex Inc., Torrance, CA) and connected to a NanoLC-1D plus HPLC system (Eksigent Technologies, LLC., Dublin, CA). Peptides were eluted with a gradient of 5 to 90 % HPLC buffer B (0.1 % formic acid in acetonitrile, *v*/*v*) in buffer A (0.1 % formic acid in water, *v*/*v*) at a flow rate of 300 nL/min over 76 min. The eluted peptides were ionized and introduced into an LTQ-Orbitrap Velos mass spectrometer (Thermo Fisher Scientific Inc., Waltham, MA) using a nanospray source. Full MS scans were acquired in the Orbitrap mass analyzer over the range m/z 300–1800 with a mass resolution of 60,000 at m/z 400. The 20 most intense peaks with charge of +2–+4 were isolated for MS/MS analysis.

### SILAC-based quantification analysis

Protein and PTM site identification and quantification were performed with MaxQuant version 1.3.0.5 software [37, 38] against Uniprot human reference protein database concatenated with reverse decoy database and protein sequences of common contaminants. Trypsin was specified as cleavage enzyme with two and four maximum missed cleavages for protein and PTM site quantification, respectively. Maximum numbers of labeled amino acids were set as 3 and 5 for protein and PTM quantification, respectively. Several variable modifications were selected: oxidation (M), acetylation (protein N-term), acetylation (K), tri-methylation (K), di-methylation (K,R), monomethylation (K,R), and specific PTM at lysine residue corresponding to each immunoaffinity purification. False discovery rate (FDR) thresholds for protein, peptide, and modification site were specified at 0.01. Minimum peptide length was set at 7 for protein quantification and 5 for PTM site quantification. Identified PTM sites with Andromeda score less than 40 or site localization probability less than 0.75 as well as site identifications from reverse or contaminant protein sequences were removed. In addition, C-terminal modified peptides were also removed unless it is located at the peptide C-terminal residue of the corresponding protein. Protein ratios were determined based on a minimum of two peptide ratios using razor and unique peptides. The final PTM site ratios were normalized by their protein ratios.

## Results

### Development of a system for titratable rates of glucose metabolism

**Fig. 1:**
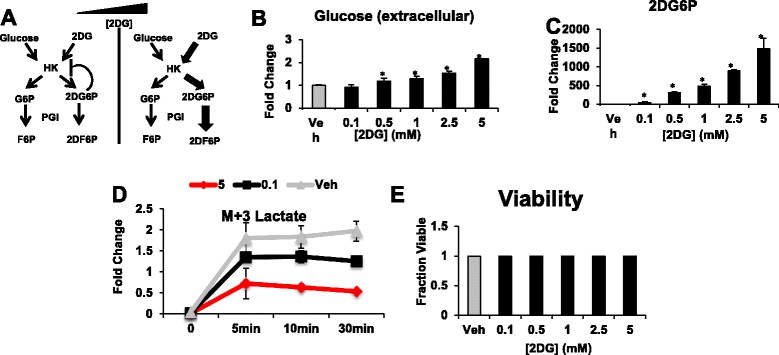
The rate of glucose metabolism and extent of metabolic reprogramming can be tuned with pharmacological manipulation. **a** Schematic of 2DG effect on glycolysis at high vs. low concentrations. **b** Relative extracellular glucose levels in response to each 2DG concentration compared to the vehicle (Veh). **c** Relative 2DG6P levels in response to 2DG treatment compared to Veh. **d** Production rate of ^13^C-labeled lactate in response to 2DG treatment. Fold change in relative amounts of lactate in media after respective treatments is depicted. **e** Cell viability test of HCT-116 cells after 6 h of growth under indicated treatments. Mean ± SEM of triplicates relative to the Veh. (*P < 0.05 relative to Veh)

To investigate quantitative mechanisms linking glucose metabolism to histone acetylation, we developed a system where the rates of glucose metabolism and as a result, levels of metabolites could be controlled in an acute manner. One possibility of a tunable system is to consider the incorporation of 2DG into cells (Fig. 1a). 2DG can affect glycolysis through multiple inhibitory mechanisms. It is taken up in cells via glucose transporters (GLUTs) where it is phosphorylated by hexokinase but cannot be further metabolized in glycolysis by phosphofructoisomerase [39]. At all concentrations, 2DG directly competes with glucose for binding and product inhibition of glucose and glucose-6-phosphate [39–42]. At higher concentrations, 2DG acquires the properties of an inhibitor of hexokinase [43–45]. At intermediate concentrations, a combination of multiple mechanisms that can affect glycolytic rate occurs [44, 46]. Thus differing concentrations of 2DG may exert unique effects on glycolysis (Fig. 1a). To capitalize on these different characteristics, we treated cells with 2DG at different concentrations with short incubation times. The short times were considered in order to mitigate potential confounding indirect effects of 2DG on cells that occur downstream of disrupting glycolysis that ensue at longer times. Notably, this timeframe is also sufficiently long for 2DG to enter cells and instantiate its mechanism of action. We therefore treated HCT116 cells with six concentrations (0, 0.1, 0.5, 1, 2.5, and 5 mM) of 2DG for a short time (6 h) that preceded growth arrest and any detectable cell death (Fig. 1e). A graded accumulation of glucose in the extracellular media was observed, as unphosphorylated glucose cannot be retained intracellularly [45] (Fig. 1b). At these differing concentrations, we next measured the phosphorylated product of 2DG, 2-deoxyglucose-6-phosphate (2DG6P), which accumulates in the cell since its negative charge precludes it from passing through cell membranes, and also observed a graded accumulation of this product as well [45] (Fig. 1c). Furthermore, we considered a kinetic flux profiling experiment using U-^13^C-glucose and detected a difference in the production rate of labeled lactate in the media further confirming that the system is affecting glycolytic flux directly at differing doses of 2DG treatment (Fig. 1d). These results indicate that a gradual change in 2DG dose results in gradual changes in glycolytic flux and the levels of metabolites both in glycolysis and across the metabolic network.

**Fig. 2:**
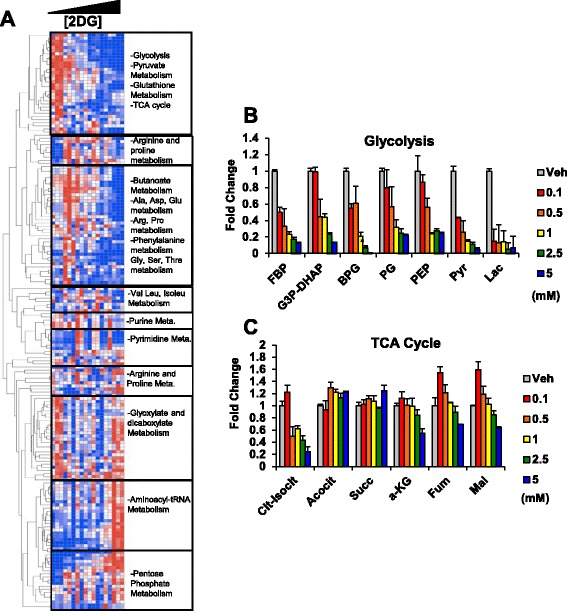
2DG treatment has a global effect on cellular metabolism. **a** Heatmap of effect of 2DG treatment on global metabolism relative to Veh. Values are row-normalized with red denoting high and blue denoting low. **b** Effect of 2DG treatment on glycolytic metabolite levels relative to Veh. **c** Effect of 2DG treatment on TCA cycle intermediate levels relative to Veh. Mean ± SEM of triplicates

To investigate how this titratable change in glycolysis globally affected cellular metabolism, we generated a profile of over 300 metabolites using a metabolomics technology that we have recently developed based on liquid chromatography coupled to high resolution mass spectrometry [34, 35, 47]. This measurement allows for the quantitation of a broad coverage of diverse metabolic pathways ranging from sugar, amino acid, to polar lipid metabolism. Across these different concentrations of 2DG, we observed graded waves of changes in metabolism involving multiple different pathways across the metabolic network (Fig. 2a). An inspection of glycolytic intermediates (Fig. 2b) revealed a graded response in the depletion of metabolites, whereas, tricarboxylic acid (TCA) cycle (Fig. 2c) intermediates displayed a more complex response. Pyruvate exhibited a qualitatively different behavior from that of the other glycolytic intermediates likely due to other sources including lactate and alanine also contributing to pyruvate biosynthesis. Other substrates such as glutamine and fatty acids are anaplerotic carbon sources in the TCA cycle that complicate these observations [10, 48]. Together, these findings indicate that glycolysis can be gradually tuned with differing levels of intermediate metabolites.

### The levels of cofactors and substrates that mediate protein acetylation are affected by glycolysis

**Fig. 3:**
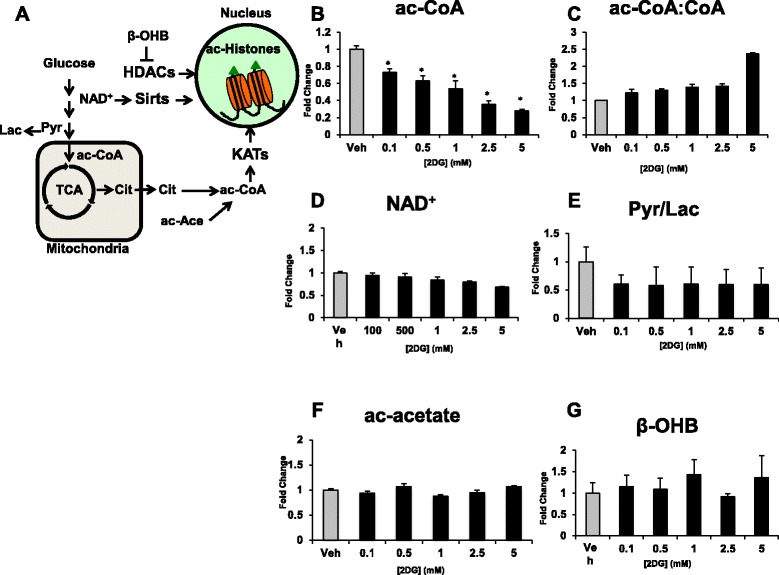
Glucose metabolism quantitatively alters substrates and co-factors for posttranslational modifications. **a** Known metabolic pathways that can affect histone acetylation. **b** ac-CoA levels in response to 2DG treatments relative to Veh. **c** ac-CoA/CoA ratio in response to 2DG treatments relative to Veh. **d** NAD^+^ levels in response to 2DG treatments relative to Veh. **e** Pyruvate/lactate ratio in response to 2DG treatments relative to Veh. **f** ac-acetate levels in response to 2DG treatments relative to Veh. **g** β-hydroxybutyrate levels in response to 2DG treatments relative to Veh. Mean ± SEM of triplicates

Having demonstrated that gradually perturbing the rate of glycolysis results in gradual differences across metabolism, we then looked across the metabolic network for the levels of metabolites that mediate protein acetylation. Several metabolites related to glycolysis and other downstream of glucose metabolism that could affect acetylation were investigated. NAD^+^ is the cofactor for sirtuins that carry out deacetylation reactions. It has also been postulated that sirtuins could be regulated by the NAD^+^/NADH ratio. Beta-hydroxybutryate (β-OHB), a ketone body, is a functional histone deacetylase (HDAC) inhibitor [29]. Also, both ac-CoA levels and the ratio of ac-CoA/CoA have been found to mediate acetylation reactions (Fig. 3a). Some of these results may be unexpected and different from what has been reported in prior studies that have used manipulations such as glucose deprivation or calorie restriction. These previous studies, however, have focused on long-term consequences of complete glycolytic inhibition as opposed to acute treatments that identify direct biochemical consequences [49].

We observed a dose-dependent gradual decrease of intracellular levels of ac-CoA (Fig. 3b) in response to 2DG treatment. We also observed a graded increase in the ac-CoA/CoA ratio (Fig. 3c). A smaller decrease in overall NAD^+^ levels was also observed (Fig. 3d). The Pyr/Lac ratio, which is a surrogate for the cytosolic NAD^+^/NADH ratio, decreased with 2DG treatment; however, this decrease was constant with all treatments (Fig. 3e). Both ac-acetate, a ketone body, (Fig. 3f) and β-OHB (Fig. 3g) were unaffected by 2DG treatment. Together, these results indicate that metabolites known to mediate protein acetylation via lysine acetyl transferases (KATs) are sensitive to glycolytic flux, whereas those known to be important for HDACs appear to be regulated by other fluxes.

### Global histone acetylation levels are determined by glycolytic flux

**Fig. 4:**
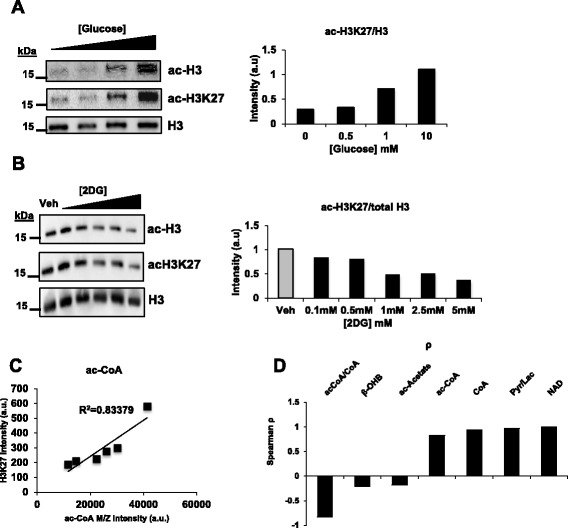
The rate of glycolysis determines histone acetylation. **a** Western Blot of Pan-acetyl-H3 and H3K27ac in response to glucose titration with quantification, total H3 used as loading control. **b** Western Blot of Pan-acetyl-H3 and H3K27ac in response to 2DG treatment with quantification, total H3 used as loading control. **c** Scatter-plot depicting correlation between ac-CoA levels and H3K27ac levels. **d** Bar graph of Spearman correlation coefficient of ac-CoA:CoA, β-OHB, ac-acetate, ac-CoA, CoA, Pyr/Lac and NAD^+^. Mean ± SEM of triplicates (*P <0.05 versus Veh)

Having observed a decrease in metabolites known to be involved in protein acetylation in response to 2DG, we next investigated whether there are corresponding changes in histone acetylation levels. We first considered whether histone acetylation would respond to glucose availability (Fig. 4a). We found that, consistent with what has been previously described [20, 21, 49], histone acetylation levels were altered in response to changes in glucose availability. Indeed, we observe a dose-dependent decrease of histone acetylation levels on global acetylated histone H3 (Ac-H3) levels as well as acetylated histone H3 at residue 27 (H3K27) levels in response to 2DG treatments (Fig. 4b). These decreases had a strong positive correlation with ac-CoA (Fig. 4c), ac-CoA/CoA (Fig. 4d), Pyr/Lac (Fig. 4d), and NAD^+^ levels (Fig. 4d). Together, these results identify the variables in the metabolic network that associate with ac-CoA and demonstrate the precise metabolite levels that result from changes in glycolytic flux that are important to regulate histone acetylation levels.

### Glycolytic flux can affect other histone acylation PTMs

We so far observed that changes in glycolytic flux induce corresponding changes in global acetylation levels and that there exist tight correlations with the levels of key metabolic variables and global histone acetylation levels. We next considered whether these changes occurred nonspecifically across the histone or whether specificity could be achieved through this mechanism as has been suggested.

**Fig. 5:**
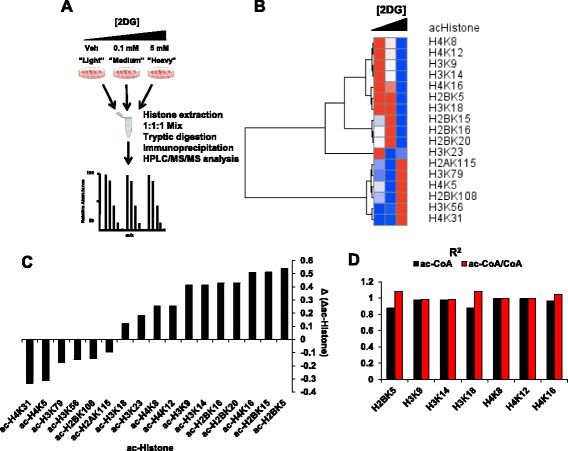
Quantitative proteomics identifies specific dose-dependent changes in histone acetylation. **a** Schematic of SILAC experiment workflow. **b** Heatmap of all detected histone acetylated residues in response to 2DG treatment. Values are row-normalized with red denoting high and blue denoting low. **c** Correlation coefficients of acetylated histones in relation to CoA and ac-CoA levels. **d** Quantification of the difference in sensitivity of ac-Histone residues to 2DG treatment

To test this hypothesis, we performed a multiplexed stable isotopic labeling by amino acids in cell culture (SILAC) experiment. Such a procedure could determine quantitative changes of specific histone acetylation sites that were modulated by glucose. We cultured cells in three media conditions containing unlabeled (^12^C) lysine (light), 4,4,5,5-D4-L-lysine (medium), and ^13^C_6_^15^N_2_-L-lysine (heavy). These conditions have extensively been shown not to affect growth rate and are minimally metabolized in cells [50, 51]. We treated these SILAC-labeled cells with 0.1 mM (low) or 5 mm (high) of 2DG and compared them to DMSO (Veh)-treated cells (Fig. 5a). We identified a total of 17 acetylation sites on histones, and after hierarchical clustering, distinct patterns of response were observed (Fig. 5b). Some residues exhibited no obvious trend in histone acetylation in response to changing rates of glycolytic flux. Others exhibited no change in total acetylation level. However, seven identified acetylation sites exhibited a graded decrease in total level in response to a decrease in glycolytic flux. Histone residues that exhibited this pattern included H3K9ac, H3K14ac, H3K18ac, H4K8ac, H4K12ac, and H4K16ac (Fig. 5b). After quantifying the difference in the change in acetylation across the three different rates of glycolysis, it was found that the levels of about 40 % (7/17) of the acetylation sites responded in a gradual quantitative manner in response to a change in glycolysis (Fig. 5c). Moreover, these sites were highly correlated to both ac-CoA levels and ac-CoA/CoA ratios (Fig. 5d). Together, these data show that manipulating metabolic flux using 2DG treatments can have specific effects on individual histone acetylation marks and that these effects can be seen at low doses.

**Fig. 6:**
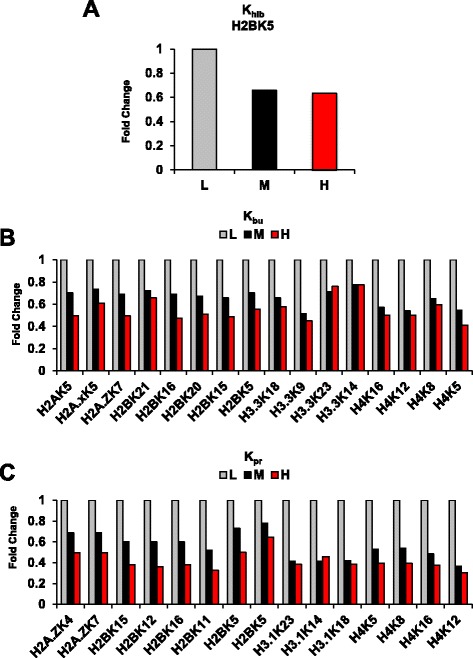
Histone acylation marks are sensitive to the rate of glycolysis. **a** 2-hydroxyisobutrylation at H2BK5 in response to 2DG treatment. **b** Detected Propionylated histone residues responses to 2DG treatment. **c** Detected Butrylated histone residues responses to 2DG treatment. L:Light (Veh), M:Medium (0.1 mM 2DG), H:Heavy (5 mM 2DG)

In addition to acetylation, it has recently come to attention that other lysine acylation modifications occur on histones. These modifications, rather than using ac-CoA as the substrate, use more chemically elaborate ac-CoA metabolites as substrates and are derived from other parts of intermediary metabolism. Whether they respond to glycolysis flux is not known. We therefore considered a further investigation of the extent that glycolysis may mediate the levels of other lysine residues. Using the previously developed multiplexed SILAC approach, we considered three additional histone lysine modifications, 2-hydroxyisobutyrylation (K_hib_) (Fig. 6a), butyrylation (K_bu_) (Fig. 6b), and propionylation (K_pr_) (Fig. 6c) totaling an additional 32 histone modifications. We found that histone propionylation (K_pr_) (Fig. 6c), histone butyrylation (K_bu_) (Fig. 6b), and histone 2-hydroxyisobutyrylation (K_hib_) (Fig. 6a) levels at all residues were gradually sensitive to glycolytic rate. Unlike lysine acetylation (K_ac_), each of these modifications appeared to quantitatively respond to changes in glycolytic flux. Together, these findings provide evidence that histone acylation exhibits specificity towards changes in glycolytic rate.

## Discussion

The molecular links between the metabolic state of cells and the status of protein acetylation have been intriguing [18, 19, 25, 52]. However, the direct quantitative biochemical interactions between metabolic flux, metabolite levels, and levels of protein acetylation have yet to be demonstrated. By considering a system that allows for controllable rates of glycolysis, this study resulted in a systematic analysis of factors in metabolism that contribute, with specificity, to acetylation levels. By considering histone modifications, this allowed for considerations of possible links between the metabolic state and cellular epigenetics. We were able to show that changes in glycolytic flux can have quantitative effects on global histone acetylation levels. This occurred first through changes in glycolytic rate having effects on the levels of metabolites in glycolysis and peripheral metabolism. Of all metabolites considered, ac-CoA and the ratio of ac-CoA to CoA appeared to have the strongest influence on histone acetylation.

Because most KATs have similar binding affinities to both ac-CoA and CoA, it has been suggested that the ac-CoA/CoA ratio may affect KAT activity as CoA can also have an inhibitory effect on some KATs [27]. Our findings demonstrate that decreasing glycolytic flux can decrease both ac-CoA and free CoA pools and that the regulation of these two pools is directly downstream of glycolysis. More importantly, our data suggests that the supply of intracellular ac-CoA has a greater effect on histone acetylation than the inhibitory effect of free CoA.

Recently, it has been shown that the selectivity of p300, a histone acetyltransferase, can be manipulated by intracellular levels of ac-CoA [53]. Furthermore, work in yeast has shown that Gcn5 is important in mediating histone acetylation and is very sensitive to intracellular ac-CoA levels as well [18]. Our findings show that many other residues not known to be catalyzed by these enzymes are also sensitive to the ac-CoA levels suggesting that such a phenomenon is likely pervasive in cells. Interestingly, it has not escaped our attention that modifications located close to or on globular histone domains seem to be more resistant to changes in glycolytic flux, whereas those on histone tails seem to be more sensitive suggesting that spatial proximity to the metabolic milieu may dictate in part those histone marks that are more dynamic. Moreover, we show that other histone acylation modifications are sensitive to glycolytic flux which likely follow similar principles.

Finally, histone Kac, Kpr, Kbu, and Khib each appear to be globally regulated by glycolytic rate and have all been associated with active gene expression. It has been shown that these PTMs utilize corresponding ac-CoA intermediates. However, it is not known that they are all connected to glycolysis. From our data, we can speculate that the decrease in free CoA restricts the ability to form the corresponding ac-CoAs necessary for these PTMs suggesting some possibility of enzymatic activity mediating these reactions. The sensitivity of these PTMs to glycolytic flux is unexpected, and further investigation is required that includes better understanding the chemical reaction mechanisms that mediate these PTMs. Their sensitivity to glycolytic flux may add to the increasing evolutionary benefits that cancer cells gain from the Warburg effect as glycolytic rates are increased in these cells by as much as 200-fold compared to normal cells. An exciting recent study has demonstrated that resistance to targeted therapy can be driven by elevated nutrient levels [54]. As abnormal metabolism is a recurrent theme in cancer, understanding its effect on chromatin biology may lead to newfound insights into tumor cell biology and the interaction between metabolism and gene regulation.

## Conclusions

Multiple functions of the Warburg Effect or the enhanced rate of glycolysis observed in tumors and proliferative tissue have been proposed. This current study provides evidence for a lesser-appreciated interpretation of the Warburg effect in that it confers direct signaling functions to cells by altering the levels of multiple key metabolites that serve as cofactors and substrates for reactions involving posttranslational modifications, notably histone acetylation, histone butyrylation, histone propionylation, and histone 2-hydroxyisobutryrylation.

## Abbreviations

Ac-CoA: acetyl coenzyme Aac-acetate aceto-acetate
CoA: coenzyme A
PTMs: posttranslational modifications
2DG: 2-deoxy-D-glucose
Veh: vehicle
2DG6P: 2-deoxyglucose-6-phosphate
β-OHB: beta-hydroxybutyrate
HDAC: histone deacetylase
KAT: lysine acetyl transferases
Ac-H3: global acetylated histone H3
SILAC: stable isotopic labeling by amino acids in cell culture
K_hib_: lysine 2-hydroxyisobutyrylation
K_bu_: lysine butyrylation
K_pr_: lysine propionylation
K_ac_: lysine acetylation
